# Inferring transcription factor collaborations in gene regulatory networks

**DOI:** 10.1186/1752-0509-8-S1-S1

**Published:** 2014-01-24

**Authors:** Sherine Awad, Jin Chen

**Affiliations:** 1 Department of Computer Science and Engineering, Michigan State University, East Lansing, Ml, USA; 2 MSU-DOE Plant Research Laboratory, Michigan State University, East Lansing, Ml, USA

## Abstract

**Background:**

Living cells are realized by complex gene expression programs that are moderated by regulatory proteins called transcription factors (TFs). The TFs control the differential expression of target genes in the context of transcriptional regulatory networks (TRNs), either individually or in groups. Deciphering the mechanisms of how the TFs control the expression of target genes is a challenging task, especially when multiple TFs collaboratively participate in the transcriptional regulation.

**Results:**

We model the underlying regulatory interactions in terms of the directions (activation or repression) and their logical roles (necessary and/or sufficient) with a modified association rule mining approach, called mTRIM. The experiment on Yeast discovered 670 regulatory interactions, in which multiple TFs express their functions on common target genes collaboratively. The evaluation on yeast genetic interactions, TF knockouts and a synthetic dataset shows that our algorithm is significantly better than the existing ones.

**Conclusions:**

mTRIM is a novel method to infer TF collaborations in transcriptional regulation networks. mTRIM is available at http://www.msu.edu/~jinchen/mTRIM.

## Background

**Figure 1:**
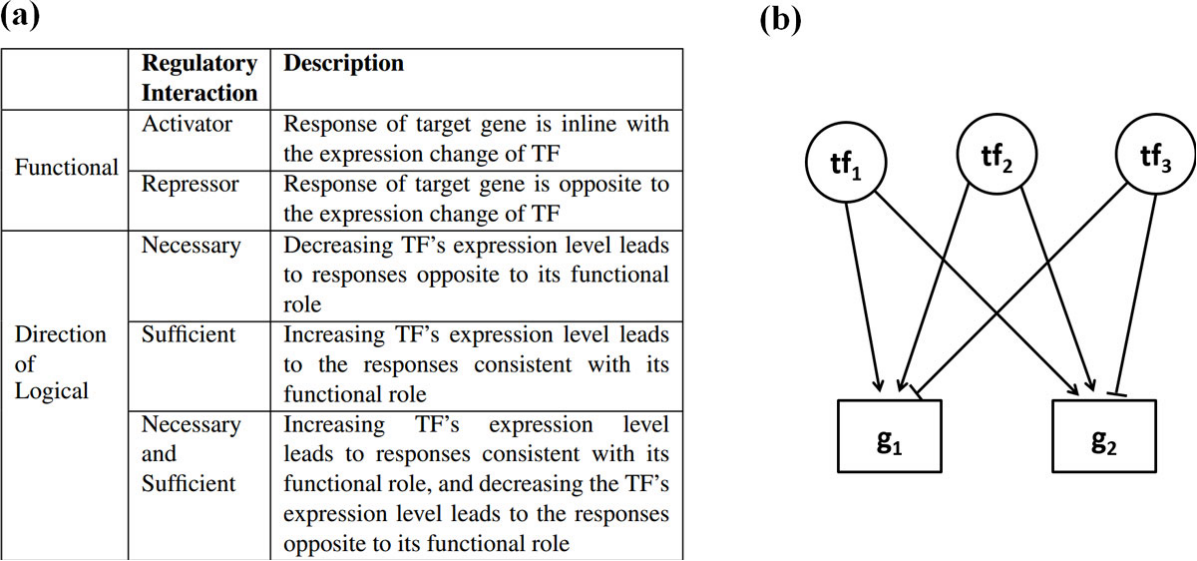
**(a) Concepts of regulatory interaction and (b) an illustrative example of regulatory interaction in a TRN, in which three TFs collaboratively co-regulate two target genes**.

The complex gene expression programs in living cells are moderated by regulatory proteins called transcription factors (TFs) [1]. In the context of a transcriptional regulatory network (TRN), a TF may act independently or collaboratively with other TFs [2], leading to complex *regulatory interactions *that influence the transcription of target genes [3,4]. A regulatory interaction includes target genes and all the TFs that control their transcriptional activities. An individual-TF regulatory interaction has been defined in terms of two properties: the TF's functional role as an activator or a repressor, and its logical role as being necessary or sufficient (see Figure 1a) [3,5]. The categories in the TF's functional and logical roles are combinable; they can be activator necessary (AN), activator sufficient (AS), or activator necessary and sufficient (ANS). For example, pheromone response elements are necessary and sufficient for basal and pheromone-induced transcription of the FUS1 gene of yeast [6]. Similarly for TFs that are repressors, they can be RN, RS or RNS [7]. In a multiple-TF regulatory interaction, a group of TFs collaborate to control the expression levels of the same target genes. The directions of all the TFs in the group, therefore, form a transcriptional regulation pattern of the target genes. Recent developments in biotechnology (such as ChIP [8] and yeast one-hybrid [9]) have been applied to uncover TF-target binding relationships [10,11] to reconstruct draft regulatory circuits at a systems level [3,4,12]. Furthermore, to identify regulatory interactions *in vivo *and consequently reveal their functions, TF single/double knockouts and over-expression experiments have been systematically carried out [13]. However, the results of many single or double-knockout (or over-expression) experiments are often non-conclusive [14], since many genes are regulated by multiple TFs with complementary functions [4]. For example, in yeast (one of the most well-studied eukaryotic organisms), 47% of genes are bound by at least two TFs [15], and approximately 73% (~4,500) of the known genes are non-essential [16], suggesting that higher order genetic variations are needed for precise inference of transcriptional regulations.

Considering the prohibitive costs and the tremendous number of possible combinations of higher-order gene knockouts, it is currently impossible for researchers to examine all of possible gene knockout combinations experimentally. One solution to this problem is to select only the TF groups that are most likely to bring about the phenotypic change. In order to accomplish this, we need to understand the interactions employed by multiple TFs (called *regulatory interactions*) to regulate their common target genes. However, this is a difficult task, because when multiple TFs simultaneously or sequentially control their target genes, a single gene responds to merged inputs, resulting in complex gene expression patterns [17,18]. The exhaustive approach requires enumerating all TF combinations, which, given the high complexity of combinatorial, is simply impractical at the whole genome level.

In our previous research [19], a Hidden Markov model was developed to relate gene expression patterns to regulatory interactions, in order to solve a relatively simpler sub-problem that considers only two TFs. To predict regulatory interactions for all possible collaborative TFs, we propose an algorithm called "mTRIM" (**m**ultiple **T**ranscriptional **R**egulatory **I**nteraction **M**echanism) in this paper. By uncovering the regulatory interactions in terms of their directions (activation or repression) and corresponding logical roles (necessary and/or sufficient) from gene expression and TF-DNA binding data, mTRIM identifies TF groups that are collaboratively responsible for target gene expressions. Such inferences may provide high-quality candidate sets for further experimentally detecting the collaborative functions of gene regulations that are largely unknown [18]. Yeang and Jaakkola [3] attempted to characterize the combinatorial regulation of multiple-TF regulatory interactions using a heuristic approach to measure how well a regulatory module fits the associated binding and gene expression data with a log-likelihood function. The regulatory module's likelihood is maximized with a greedy approach by incrementally adding genes to the module and monitoring the predictions of the regulatory interactions for optimality. However, this incremental approach does not study the functions of the TFs simultaneously because of the scalability issue introduced by the greedy search. This method also uses a probability-based approach to calculate the significance of the combinatorial property of TFs, determined by the gap of likelihood scores between their model and a model built on randomized data in the entire time frame. However, as stated in [4], a TF usually functions at specific "activation time points" instead of throughout the entire time course, meaning that the identification of regulatory interaction modules should be focused on activation time-points rather than the entire time frame.

To derive dynamic regulatory networks that associate TFs with target genes at their activation time-points, an algorithm called DREM was proposed [4]. DREM integrates time-series gene expression data and protein-DNA binding data to build a global temporal map, in order to uncover transcriptional regulatory events leading to the observed temporal expression patterns and the underlying factors that control these events during a cell's response to stimuli. The method mainly works by identifying bifurcation time-points where the expression of a subset of genes diverges from the rest of the genes. The bifurcation points are then annotated with the TFs regulating these transitions, which result in a unified temporal map. The method can therefore facilitate the determination of the time when TFs are exerting their influence, and assigns genes to paths in the map based on their expression profiles and the TFs that control them. Unlike the method by Yeang and Jaakkola [3], DREM's ability to derive dynamic maps that associate TFs with the genes they regulate and their activation time-points has indeed led to better insights for the regulatory module being studied. However, DREM does not infer the logical roles of the TFs (*i.e*., whether a specific TF is necessary or sufficient for regulating a set of target genes). Such knowledge is extremely useful for designing high-order genetic variation experiments to understand the complex regulatory mechanisms of biological processes.

TRIM is an HMM based model which was developed to infer the collaboration of at most two TFs that regulate the same target genes. In the HMM, the functions of a TF are hidden states. The model starts with random priors, and then is iteratively trained using EM till convergence. Since each possible function of a TF is a node in the HMM, there are four nodes (AS, AN, RS, and RN) for each TF. With the design of HMM (and the limited training data), the number of TFs TRIM can handle is limited.

The enumeration of all TF combinations is clearly a NP problem. Therefore, we focused on the most important biological problem (i.e., 2-TF combination) and therefore "hardcoded the problem in TRIM. In this paper, we solve the efficiency problem by developing an association rule mining algorithm which is capable to handle a large amount of data with high-level combinations.

In this paper, we propose a new model mTRIM for inferring regulatory interactions for multiple TFs with an EM-based Bayesian inference approach [20,21] and a modified bottom-up association rule mining method. Experimental results evaluated with yeast genetic interactions, TF knockouts and a synthetic dataset shows that our algorithm is significantly better than the existing ones.

## Methods

mTRIM is developed to efficiently infer regulatory interactions for all possible collaborative TFs in a TRN. The feasibility is achieved in two steps. First, an EM-based Bayesian inference approach is developed to identify all the significant individual TF regulatory interactions, meaning that individual TFs that can regulate the target genes independent to the existence of other TFs. For the TFs which require collaborations with other TFs to drive the target genes, or are actually non-deterministic (meaning lack of clear evidence of regulation), their p-values are insignificant. They are considered as the inputs of the second step.

Second, in order to identify the collaboration of *k *TFs (*k *≥ 2), *i.e*., *k*-TF regulatory interaction, a bottom-up association rule mining approach is developed. While the significant TF groups are reported to the users, the insignificant ones are joined with each other to mine (*k *+ 1)-TF regulatory interactions. It should be noted that unlike the conventional association rule mining which seeks the longest possible patterns, mTRIM outputs the shortest significant results, in that the goal of mTRIM is to discover the smallest group of TFs that can regulate the target genes, so that biological experiments with high-order genetic variations can be subsequently carried out for the understanding of the behavior of TRNs. In terms of time complexity, consider a candidate *k*-TF regulatory interaction $I=<h_{tf_{1}},...,h_{tf_{k}}>\Rightarrow h_{g}$. The algorithm computes *AfnScore *and p-values of all of the subsets, *I *- {*tf_j_*} (∀*j *= 1, 2, ..., *k*). If one of them is significant, *I *is immediately pruned. Hence the time complexity is *O*(*k*) for each candidate *k*-TFs regulatory interaction. Every merging operation requires at most *k *- 2 equality comparisons. In the best-case scenario, it produces a viable candidate *k*-TF interaction. In the worst case, the algorithm merges every pair of infrequent (*k *- 1)-TF candidates. Therefore, the overall cost of merging candidates is between $\sum_{k=2}^{\left|TF\right|}\left(k-2\right)|P_{k}|\;\text{and}\sum_{k=2}^{\left|TF\right|}\left(k-2\right)|P_{k-1}{|}^{2}$, where *P_k _*is the candidate set of *k*-TF regulatory interactions. To improve the algorithm efficiency, a hash tree is constructed for the storage and quick access to all of the candidates. Because the maximum depth of the hash tree is *k*, the cost for populating the hash tree of candidates is $O\left(\sum_{k=2}^{\left|TF\right|}k|P_{k}|\right)$. During candidate pruning, it is required to verify whether the *k *- 1 subsets of every candidate *k*-TF regulatory interactions are significant. Since the cost for looking up an item in a hash tree is *O*(*k*), the time complex of candidate pruning step is $O\left(\sum_{k=2}^{w}k\left(k-2\right)|P_{k}|\right)$.

### Concepts

A TRN can be represented as a directed graph in which each node is a TF or a gene, and each edge pointing from a TF to a gene represents a regulation relationship between them. In many organisms, in-depth transcriptome analysis has revealed the modular architecture of gene expression [22]. A regulatory module is a self-consistent regulatory unit *R*(*TF*, *G*, *I*) representing a set of co-expressed genes *G *= {*g*_1_, *g*_2_, ..., *g_n_*} regulated in concert by a group of TFs in *TF *= {*tf*_1_, *tf*_2_, ..., *tf_m_*} that govern the target genes' behaviors via regulatory interaction *I *[5]. An example of the regulatory module is shown in Figure 1b.

A regulatory interaction $I=<{h_{tf}}_{{}_{1}},\;\ldots ,\;h_{tf_{i}},\;\ldots ,\;h_{tf_{m}}>\Rightarrow h_{g}$ (which is the final output of mTRIM) is defined as a set of TFs {*tf*_1_, ..., *tf_m_*} co-regulating a set of genes {*g*_1_, ..., *g_n_*}, where $h_{tf_{i}}$ is the behavior of TF *i*; *h_g _*is the behavior of all the target genes in *R*, and *h_x _*∈ {↑, ↓, -}, meaning up-express, down-express and no change respectively. For example, if *tf*_1 _↑ and *tf*_2 _↓ always cause the target genes *g*_1 _and *g*_2 _to be up-regulated, the regulatory interaction is <*tf*_1 _↑, *tf*_2 _↓> ⇒ *g *↑. For individual regulatory interactions, *I *∈ {*AN*, *AS*, *RN*, *RS*, *ANS*, *RNS*}. In this work, we assume that a regulatory interaction is consistent in the context of transcriptional control as long as the experimental conditions are unchanged. Note that binaries gene expression values are used in mTRIM, since TF activity is not always proportional to its mRNA abundance [23].

### mTRIM Step 1. Inferring individual regulatory interactions

To solve a relatively easier problem of inferring the regulatory interactions for each individual TF and to prepare input for multi-TF regulatory interaction inference, an EM-based Bayesian inference algorithm has been developed [20,21].

To define the probabilities in Eq. 2 and Eq. 3, we followed the definitions in [20]. Eq 2 represents the prior probability of the interaction model *I_m_*, and Eq 3 represents the probability of gene expression correlation between TFs and targets given the interaction model *I_m_*. In the Bayesian model, the training dataset is a matrix that contains gene expression levels of TFs and their targets, from which Γ(*I_m_*) is estimated using Eq 4. And then, the likelihood is calculated using Eq 3. The prior probabilities are randomly assigned initially. In each iteration, the posterior probabilities and the frequency of *I_m _*are updated. The iteration will continue till the posterior probabilities converge.

Let *Pos *be the posterior probability of a TF *tf_m _*to have a specific regulatory interaction *I_m _*in regulatory module *R_k_*, where *I_m _*∈ {*AN*, *AS*, *RN*, *RS*} (ANS and RNS will be discussed later). To infer *Pos*, both the prior probabilities *Pri *and the likelihood *Lk *of the same TF need to be computed, given that:

(1) $$Pos\left(tf_{m},\;R_{k},\;I_{m}\right)=Pri\left(I_{m}\right)\times Lk\left(tf_{m},\;R_{k},\;I_{m}\right)$$

where *Pri*(*I_m_*) is the prior probability of regulatory interaction *I_m _*(defined in Eq 2) and the likelihood *Lk*(*tf_m_*, *R_k_*, *I_m_*) is defined in Eq 3.

The prior probability *Pri*(*I_m_*) captures how likely a given interaction *I_m _*exists given the background of all of the other TFs:

(2) $$Pri\left(I_{m}\right)=\frac{fre\left(I_{m}\right)}{\left|R|\times |TF\right|}$$

where *fre*(*I_m_*) is the frequency of regulatory interaction *I_m _*in all of the regulatory modules, |*R*| is the number of the regulatory modules, |*TF*| is the number of TFs, and *I_m _*∈ {*AS*, *RS*, *AN*, *RN*}.

Given the definition of a regulatory interaction, the likelihood *Lk*(*tf_m_*, *R_k_*, *I_m_*) indicates how likely *tf_m _*in *R_k _*has regulatory interaction *I_m_*, which is defined by the expression level changes of the TF and its targets:

(3) $$Lk\left(tf_{m},\;R_{k},\;I_{m}\right)=\frac{\sum_{t=1}^{T-1}\sum_{n=1}^{\left|G\right|}\Gamma \left(I_{m}\right)}{\sum_{r=1}^{\left|R\right|}\sum_{m=1}^{\left|TF\right|}\sum_{t=1}^{T-1}\sum_{n=1}^{\left|G\right|}\Gamma \left(I_{m}\right)}$$

where *T *is the number of time-points in the training data, |*G*| is the number of genes in regulatory module *R_k_*, and Γ(*I_m_*) is defined as:

(4) $$\Gamma \left(I_{m}\right)=\left\{\begin{array}{cc} 1 & \text{if}\;I_{m}=\;\text{AS}\;\text{and}\;\left(tf_{m}↑\;\text{and}\;g↑\right),\;or\\ & \text{if}\;I_{m}=\;\text{RS}\;\text{and}\;\left(tf_{m}↑\;\text{and}\;g↓\right),\;or\\ & \text{if}\;I_{m}=\text{AN}\;\text{and}\;\left(tf_{m}↓\;\text{and}\;g↓\right),\;or\\ & \text{if}\;I_{m}=\;\text{RN}\;\text{and}\;\left(tf_{m}↓\;\text{and}\;g↑\right)\\ 0 & \text{otherwise} \end{array}\right)$$

An expectation-maximization (EM) algorithm is adopted to maximize the posterior probabilities *Pos*(*tf_m_*, *R_k_*, *I_m_*). The EM model is initialized with each TF assigned a random regulatory interaction. In the expectation step, we compute the likelihood of each TF to be a specific interaction using Eq 3. Consequently, the posterior probabilities of interactions for every TF is updated with Eq 1. As a result, each TF is assigned with the regulatory interaction with the highest posterior probability. In the maximization step, we maximize the scoring function $S\left(R_{k}\right)=\sum_{m=1}^{\left|TF\right|}\sum_{n=1}^{\left|G\right|}\Gamma \left(I_{m}\right)$ for each regulatory module *R_k_*, which measures how the interaction of each TF in *R_k _*matches the target gene expression changes. Note that in the iteration the priors are updated but the likelihoods are constant.

Finally, in order to determine whether *I_m _*is "necessary and sufficient" (ANS and RNS) or "no decision", the following strategy is adopted: if none of the posterior probabilities are significant, the output is "no decision"; if the probabilities of both *N *and *S *states are significant, and there is no significant difference between them, the output is *ANS *or *RNS *depending on the target gene expression direction; otherwise the output is the regulatory interaction with the highest posterior probability.

An illustrative example is shown in Figure 1b, in which *tf*_1_, *tf*_2 _and *tf*_3 _regulate target genes *g*_1 _and *g*_2_, and they all belong to the same regulatory module *R_k_*. With the gene expression changes in Table 1, we start with equal prior probabilities, *i*.*e*., *Pri*(*AS*) = *Pri*(*RS*) = *Pri*(*AN*) = *Pri*(*RN*) = 0.25, so *Lk*(*tf*_1_, *R*_k_, *AN*) = 12/26 = 0.461, (Eq 3). After 10 iterations, in the expectation step, *Pri*(*AN*) is updated to 0.70 (Eq 2), hence *Pos*(*tf*_1_, *R_k_*, *RS*) = 0.70 × 0.461 = 0.323 (Eq 1). In the maximization step, we have <*tf*_1 _↓> =>*g *↓, because the maximum posterior probability is assigned to *AN *with p-value 0.05 (see Table 2 row 1).

**Table 1:** Illustrative example of time-series gene expression data for the genes in Figure 1b.

	*t*_0_	*t*_1_	*t*_2_	*t*_3_	*t*_4_	*t*_5_	*t*_6_	*t*_7_	*t*_8_	*t*_9_	*t*_10_	*t*_11_
*tf*_1_	↑	↑	↑	↑	↑	↑	↓	↓	↓	↓	↓	↓

*tf*_2_	↑	↑	↑	↑	↑	↑	↑	↑	↑	↑	↑	↑

*tf*_3_	↓	↓	↓	↓	↓	↓	↓	↓	↓	↓	↓	↓

*g*_1_	↑	↑	↑	↑	↑	↓	↓	↓	↓	↓	↓	↓

*g*_2_	↑	↑	↑	↑	↑	↓	↓	↓	↓	↓	↓	↓

**Table 2:** Illustrative example of regulatory interaction identification on the TRN in Figure 1b.

	Regulatory Interaction	*AfnScore*	p-value
*I*_0_	<*tf*_1 _↓>⇒ *g *↓	-	0.05

*I*_1_	<*tf*_1 _↑, *tf*_2 _↑>⇒ *g *↑	0.347	0.06

*I*_2_	<*tf*_2 _↑, *tf*_3 _↓>⇒ *g *↑	0.173	0.09

*I*_3_	<*tf*_1 _↑, *tf*_3 _↓>⇒ *g *↑	0.347	0.06

*I*_4_	<*tf*_1 _↑, *tf*_2 _↑, *tf*_3 _↓>⇒ *g *↑	0.347	0.04

### mTRIM Step 2. Mining multiple-TF regulatory interactions

Besides the individual TF regulatory interactions, a significant portion of TFs collaboratively work together to regulate the same target genes. In order to identify these multiple-TF regulatory interactions, a new association rule mining approach has been developed. Instead of using the concepts of support and confidence that are commonly used in a conventional association rule mining application [24], we define an affinity scoring function (called *AfnScore*) according to the gene expression agreement between the TF groups and their target genes, to meet the biological meaning of a multiple-TF regulatory interaction (see Section Background). Mathematically, *AfnScore *of each candidate regulatory interaction $I=<h_{tf_{1}},\;h_{tf_{2}},\;\ldots ,\;h_{tf_{m}}>\Rightarrow h_{g}$ is calculated with:

(5) $$AfnScore\left(I\right)=\frac{P\left(h_{tf_{1}},h_{tf_{2}},\ldots ,h_{tf_{m}},h_{g}\right)*P\left(h_{g}\right)}{P\left(h_{tf_{1}},h_{tf_{2}},.\;.\;.,h_{tf_{m}}\right)}$$

where *P*(*x*) is the number of times that *x *appears in the given time series gene expression dataset divided by the product of the total number of time points and the total number of target genes. The p-value of each candidate regulatory interaction is computed by considering the distribution of *AfnScore *for the regulatory interactions with the same number of TFs. Only the candidate interactions with p-values smaller than 0.05 are reported to the user. Specifically, if all the TFs in *I *are up-regulated, the TFs are "sufficient"; if they are all down-regulated, the TFs are "necessary"; otherwise, each TF acts differently to drive the target genes to the same direction.

**Figure 2:**
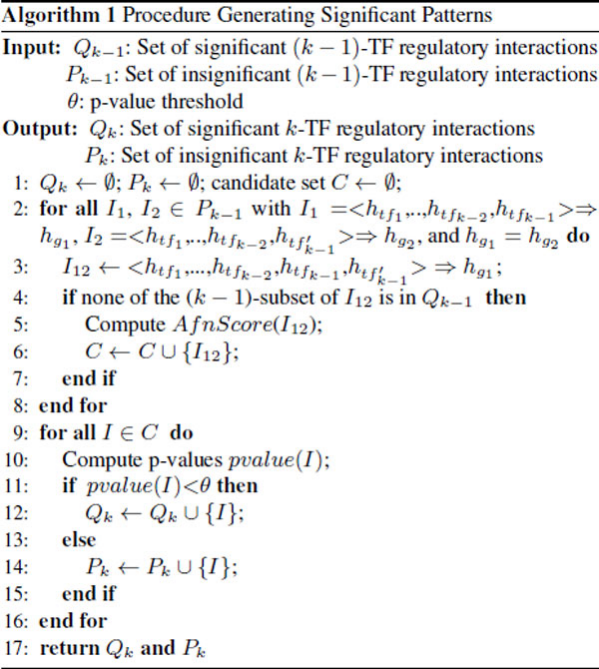
**Procedure of identifying significant multiple-TF regulatory interactions**.

To identify all the significant *k*-TF regulatory interactions, the new association rule mining algorithm starts with an empty set *Q_k _*and all the insignificant (*k *- 1)-TF interactions saved in *P_k-1 _*(see pseudocode in Figure 2 line 1). For interactions $I_{1}=<h_{tf_{1}},\;\ldots ,\;h_{tf_{k-1}}>\Rightarrow h_{g}$ and $I_{2}=<h_{tf_{1}}^{\prime },\;\ldots ,\;h_{tf_{k-1}}^{\prime }>\Rightarrow h_{g}^{\prime }$in *P_k-1_*, we combine them and compose a new interaction *I*_12 _(line 3), if *I*_1 _and *I*_2 _are combinable. We define that *I*_1 _and *I*_2 _are combinable if and only if they satisfy the conditions that $h_{g}=h_{g}^{\prime },\;h_{tf_{i}}=h_{tf_{i}}^{\prime }$ (for *i = *1, 2, .., *k - *2) and $h_{tf_{k-1}}\neq h_{tf_{k-1}}^{\prime }$. If none of the (*k - *1)-TF subsets of *I*_12 _is significant (line 2-8), *I*_12 _is added to candidate set *C *and its *AfnScore *is computed. Finally, we compute p-values for all of the *k*-TF candidates in *C *using t-test, report all of the significant regulatory interactions to the user, and save all the insignificant ones *P_k _*to for the identification of the (*k *+ 1)-TF regulatory interactions (line 9-17).

For an illustrative example, there are 40 possible multiple-TF regulatory interactions in the regulatory module shown in Figure 1b. Using the time-series gene expression data in Table 1 all the 2-TF regulatory interaction candidates are screened and their p-values are computed (see Table 2 row 2-4). Since none of the 2-TF regulatory interaction candidates is significant, a 3-TF interaction *I*_4 _=<*tf*_1 _↑, *tf*_2 _↑, *tf*_3 _↓>=>*g *↑ is generated by merging *I*_2 _and *I*_3_. The *AfnScore *of *I*_4 _is ((10/24) * (10/24))/(12/24)) = 0.347 and its p-value is 0.04 (see Table 2 row 5). Based on *I*_0 _and *I*_4_, we conclude that the target genes *g*_1 _and *g*_2 _are induced by the up-expression of *tf*_1 _and *tf*_2 _and the down-expression of *tf*_3_, and the same target genes are repressed by the down-expression of *tf*_1_.

## Experimental results

mTRIM was applied on two independently-constructed yeast transcriptional regulatory networks (the Harbison dataset [15] and the Reimand dataset [12]) to identify regulatory interactions. For performance comparison, DREM v3.0 [17] and TRIM [19] were both applied on the same datasets. We did not compare mTRIM with Yeang's method [3] because the latter's objective is to build a reliable TRN instead of predicting regulatory interactions. We evaluated these methods systematically with three independent sources: single TF knockouts [16] for individual regulatory interactions, genetic interactions (GI) [25] for 2-TF regulatory interactions and synthetic data for high-order regulatory interactions.

Using the EM-Based Bayesian inference approach, 658 significant individual regulatory interactions were mined in the Harbison dataset and 164 significant ones were mined in the Reimand dataset (Table 3). The results show that while many individual TFs drive target genes' behaviors, it is clear that most of them (4,414 in the Harbison dataset and 1,539 in the Reimand dataset) are "no decision". It indicates that a large proportion of TFs need to work collaboratively with other TFs.

**Table 3:** The number and type of the regulatory interactions for individual TFs predicted by mTRIM.

Dataset	Necessary		Sufficient		Necessary & Sufficient		No Decision
	**Activator**	**Repressor**	**Activator**	**Repressor**	**Activator**	**Repressor**	

**Harbison**	194	184	118	162	29	69	4414

**Reimand**	22	43	42	32	7	18	1543

Multiple-TF regulatory interactions were inferred with a new association mining algorithm. In total, 670 regulatory interactions with multiple TFs were discovered (Table 4). The results show that at most 6 TFs collaboratively regulate the same target genes. All the TF combinations with more than 6 TFs are either insignificant or have a significant subset. The whole experiments finished in 30 minutes on a high performance computer cluster.

**Table 4:** Number of the multiple-TF regulatory interactions identified by mTRIM.

Dataset	2-TF	3-TF	4-TF	5-TF	6-TF
**Harbison**	350	61	82	43	10

**Reimand**	95	15	7	7	0

### Data preparation

Yeast ChlP-chip binding data [15] was downloaded from http://younglab.wi.mit.edu/regulatory_code, and a p-value cutoff of 0.001 was applied (the same threshold used in [4]) to obtain the Harbison dataset. It contains 169 TFs, 2,864 target genes and 6,253 TF-DNA bindings. Next we applied the same statistical approach as in [12] to filter the union of the yeast ChlP-chip binding data [26] and the binding-site predictions [27,28] to generate the Reimand dataset with 2,230 TF-DNA binding relationships between 268 TFs and 1,509 target genes. To obtain the regulatory modules in the TRNs, all the target genes were clustered based on their gene expression values with Cluster 3.0 (specifically, k-means), which uses Pearson correlation coefficient for gene similarity metric [29], resulting in 50 clusters. The clusters are then evaluated with Gene Ontology enrichment analysis using Bingo [30], and unenriched clusters are discarded. To construct regulatory modules from the clustering results, the target genes that are regulated by the same TFs were partitioned if they are not in the same cluster. Finally, 2,172 and 1,031 regulatory modules were obtained in the Harbison and Reimand networks respectively. The distribution of genes and regulatory modules (Figure SI and Table S2 in Additional file 1) reveal that many genes are bound by multiple TFs.

To identify the individual and collaborative regulatory interactions in the above datasets, three widely used time-series microarray datasets (alpha, CDC28 and elu) from yeast cell cycle studies were collected [31] as training data. These datasets contain 49 time points in total. In these experiments, yeast cells were first synchronized to the same cell cycle stage, released from synchronization, and then the total RNA samples were taken at even intervals for a period of time (Table SI in Additional file 1). In order to decide whether a gene is significantly up or down regulated, a gene expression change cutoff of 0.35 was applied (the same threshold used in [19]).

To evaluate the individual regulatory relations, single-TF knockout microarray data were collected [16], and a p-value cut-off of 0.05 (as used in [16]) was applied to determine whether a gene is significantly affected by a TF knockout. To evaluate the 2-TF regulatory interactions, we downloaded the SGA genetic interaction dataset [25], which is composed of 1,711 queries crossed to 3,885 array strains. Of the 1,711 queries, 1,377 are deletion mutants of non-essential genes and 334 are essential gene alleles. The SGA dataset contains 762,146 genetic interactions. Two genes are genetically interacted if mutations in both of them produce a phenotype that is significantly different to each mutation's individual effects. In a 2-TF regulatory interaction, if TFs collaboratively regulate the same target genes, the down-regulation of both TFs should have a significantly different phenotype as the down regulation of each individual TF. Therefore, such TF pairs should have a significant p-value in the GI dataset. To evaluate the high-order multiple-TF regulatory interactions, a synthetic binding network were built, which contains 11 TFs, 17 target genes and 58 regulation/binding relationships. The network also contains two feed forward loops. Corresponding time-series gene expression data containing 500 time-points were randomly generated with 10% or 40% noise rate.

### Evaluation 1. Single TF knock-outs

We used the single TF knockout microarray data to evaluate the performance of mTRIM on individual TF regulatory interaction predictions in terms of the identification of "necessary" TFs (*i.e*., if the expression values of the target genes are significantly changed when the TF is knocked out). For the Harbison dataset, the prediction precision of mTRIM is 94.44%, higher than the results of TRIM (82.50%). Using the Reimand dataset, mTRIM has a precision of 91.94%, significantly higher than the results of TRIM (61.54%). DREM is not compared since it does not predict "necessary" TFs.

### Evaluation 2. Genetic interaction

**Figure 3:**
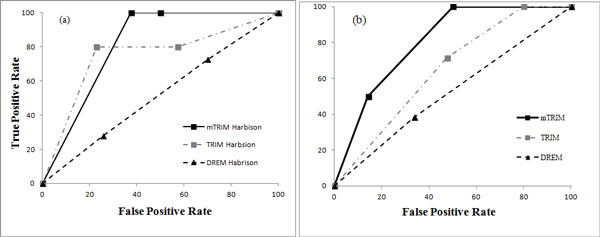
**Evaluation of the 2-TF regulatory interactions using genetic interactions on (a) Harbison dataset and (b) Reimand dataset**.

In a regulatory module with two TFs, if both TFs collaborate to regulate the same target genes, the down- regulation of both TFs should have significantly different phenotypes from the down-regulation of each individual TF. Therefore, such TF pairs should have a significant p-value in the GI dataset. To this end, for the pairs of TFs that are predicted by mTRIM to work collaboratively, we adopted the GI dataset [25] for evaluation. Figure 3 (a) and 3 (b) shows the Receiver Operating Characteristic curve (ROC) of mTRIM, TRIM and DREM on Harbison dataset and Reimand dataset respectively. For Harbison dataset, the area under curve (AUC) of mTRIM is 0.81, much higher than the AUC of DREM (0.51) and TRIM (0.75). For Reimand dataset, the AUC of mTRIM is 0.80, higher than DREM (0.52) and TRIM (0.64). In addition, to explore whether the performance of mTRIM is sensitive to parameter settings, we altered its parameters systematically. For the Harbison dataset, Figure S2 in Additional file 1 shows the AUC values with different gene expression cutoffs, GI cutoffs, and p-value cutoffs of *AfnScore *respectively. Similarly, for Reimand dataset, Figure S2 in Additional file 1 shows the varying of the AUC values using different thresholds. These show that our method is robust with the GI cutoff and p-value cutoff of *AfnScore*, although its performance gradually decreases with the increase of gene expression cutoffs.

### Evaluation 3. Synthetic transcriptional regulatory networks

**Figure 4:**
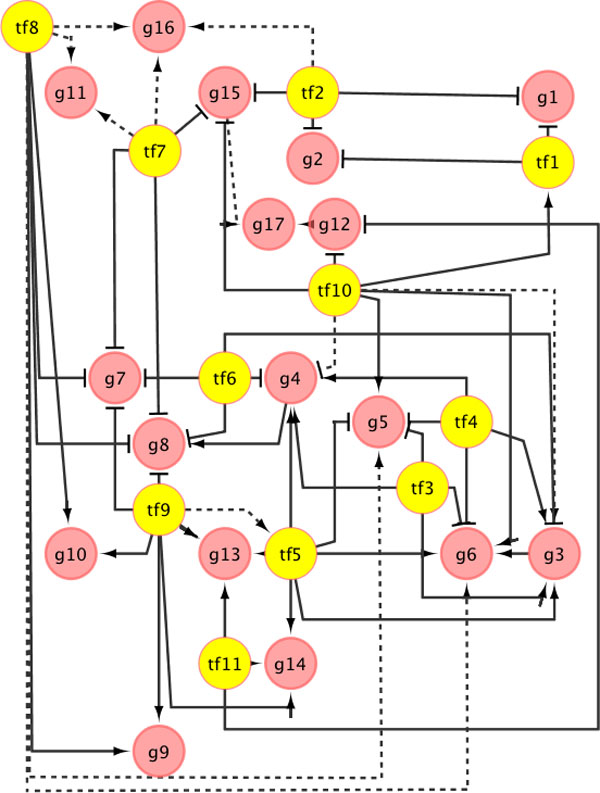
**A synthetic transcriptional regulatory network, in which the solid lines represent transcriptional regulations and the dotted lines represent TF-DNA bindings only (meaning binding but not regulation)**.

A synthetic transcriptional regulatory network was generated to evaluate the performance of mTRIM in detecting high-order multiple-TF regulatory interactions (see Figure 4). The synthetic network has 28 nodes (11 TFs and 17 target genes) and 58 edges, in which the solid line represents a real transcriptional regulation and 12 (20.69%) dotted lines represent TF-DNA bindings but no regulation. The dotted lines were added to the network in order to test the precision of mTRIM. For the synthetic network, two time series gene expression datasets with 500 time-points were generated. In order to test the robustness of mTRIM, we repeated the simulation test twice with different rates of noises added to the simulated gene expression data sets.

**Figure 5:**
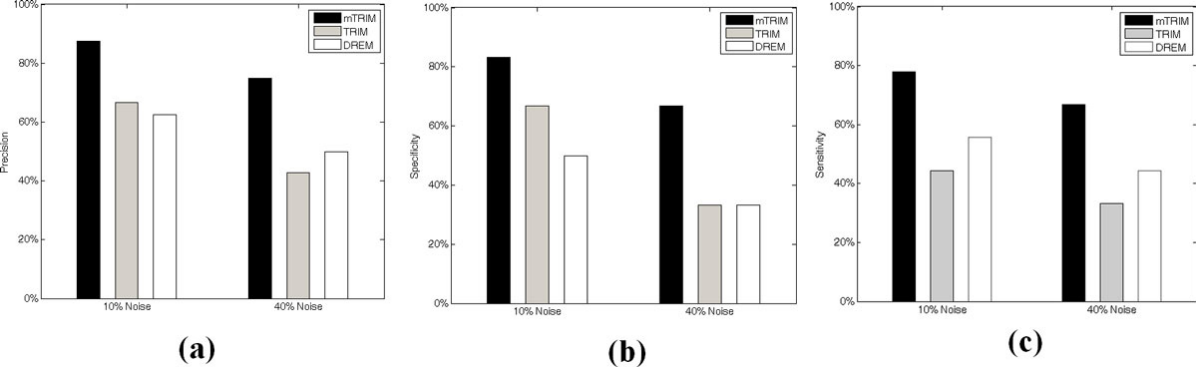
**Comparing mTRIM with DREM and TRIM on two sets of synthetic data with different noise rates**.

A comparison between all the three algorithms (see Figure 5) indicates that the performance of mTRIM is constantly the best on precision, specificity and sensitivity (equivalent to recall). Precisely, the precision of mTRIM is 87.5%, while the precisions of DREM and TRIM are 62.5% and 66.67% respectively. The recall of mTRIM is significantly higher than TRIM because it identified 4 out of 5 regulatory interactions with more than two TFs, while TRIM, because of the scalability issue, cannot find any regulatory interactions with more than two TFs. It also shows that mTRIM is less sensitive to the change of the noise rates from 10% to 40% in the gene expression data than the other two algorithms.

### Case studies

**Figure 6:**
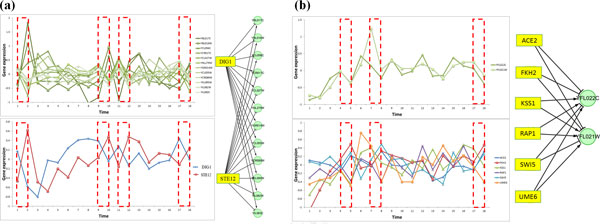
**Case studies of regulatory interactions in the Harbison dataset**: (a) a 2-TF (STE12 and DIG1) regulatory interaction controlling 12 genes; (b) a 6-TF regulatory interaction, which reveals the collaboration of multiple pathways.

In Figure 6a, a 2-TF regulatory interaction that controls 12 target genes were found in the Harbison dataset. The yellow colored nodes are TFs and the green colored nodes are their target genes. The red boxes of the dotted lines represent the time points when the TFs collaborate with each other to regulate the target genes. STE12 (which is activated by a MAP kinase signaling cascade) activates genes involved in mating or pseudohyphal/invasive growth pathways. DIG1 is the MAP kinase-responsive inhibitor of STE12. The target genes are enriched in "response to pheromone" (6 genes), "growth" (3 genes) and so on. The collaboration between STE12 and DIG1 on cell growth was captured by mTRIM successfully. Another interesting result found in the same dataset is a 6-TF regulatory interaction (Figure 6b). All the six TFs are well-characterized in yeast but are considered to function in different pathways. Our finding connects the distinct biological processes, revealing potential TF collaborations at the transcription level.

## Conclusion

Revealing the mechanisms of the transcriptional regulatory programs in TRNs is essential for understanding the complex control by which genes are expressed in living cells. The inference of collaborative protein-DNA functions helps paving the critical path for new drug development. In this work, we identify the *regulatory interactions *between TFs and target genes with mTRIM, an integration of an EM-based Bayesian inference and a new association rule mining approach built on a set of basic constraints that relate gene expression patterns to regulatory interactions. mTRIM is not limited by the number of TFs. The experimental results show that mTRIM is clearly better than the existing algorithms. Since it is difficult to obtain the ground truth for algorithm performance evaluation on real data, we generated two sets of synthetic data and used them to validate the results of our algorithm. In our future work, we will use third-party biological evidences including multiple TF knockouts, metabolic pathways, protein-protein interactions, etc., for biological validation. In our future work, we would like to extend this work by including extra data in addition to wild-type gene expression datasets. For example, since miRNA can degrade the genes induced by certain TFs [32], we will consider miRNA-target bindings and miRNA expressions, aiming to understand how miRNAs and TFs collaborate to regulate target gene expressions.

## Competing interests

The authors declare that they have no competing interests.

## Authors' contributions

JC conceived the project. Sa and JC designed the algorithm and experiments. SA implemented the algorithm and finished the experiments.

## Supplementary Material

Additional file 1 **Supplementary Materials for Awad et al. Figures SI, S2, Table SI, and S2**. This file contains Figures SI, S2, Tables SI and S2. Click here for file

## Declarations

The publication costs for this article were funded by the corresponding author's institution.

This article has been published as part of *BMC Systems Biology *Volume 8 Supplement 1, 2014: Selected articles from the Twelfth Asia Pacific Bioinformatics Conference (APBC 2014): Systems Biology. The full contents of the supplement are available online at http://www.biomedcentral.com/bmcsystbiol/supplements/8/S1.
