# Supplementary material for: Inferring transcription factor collaborations in gene regulatory networks
Source: BMC Syst Biol. 2014 Jan 24;8(Suppl 1):S1. doi: 10.1186/1752-0509-8-S1-S1 (PMC4080427; doi:10.1186/1752-0509-8-S1-S1)
Supplement: Additional file 1 — Supplementary Materials for Awad et al. Figures SI, S2, Table SI, and S2. This file contains Figures SI, S2, Tables SI and S2. [file 1752-0509-8-S1-S1-S1.pdf]

Additional file 1: Supplementary Materials for Awad et al. Figures S1, S2, Table S1, and S2.

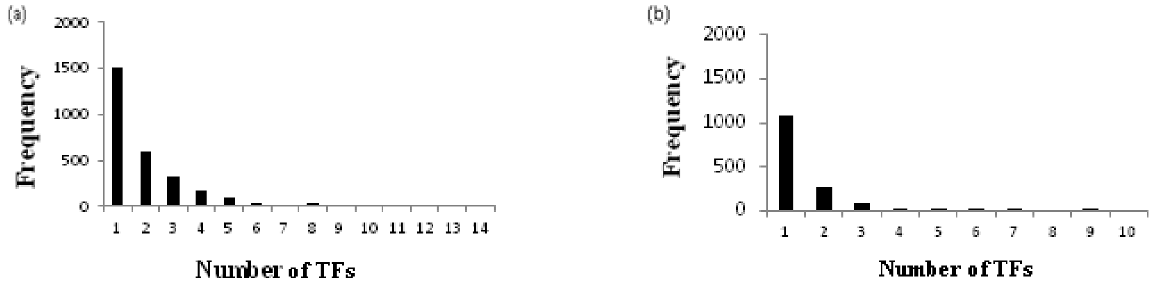

Figure S1: Distribution of the genes bound by the same TFs for the Harbison (a) and the Reimand (b) datasets.

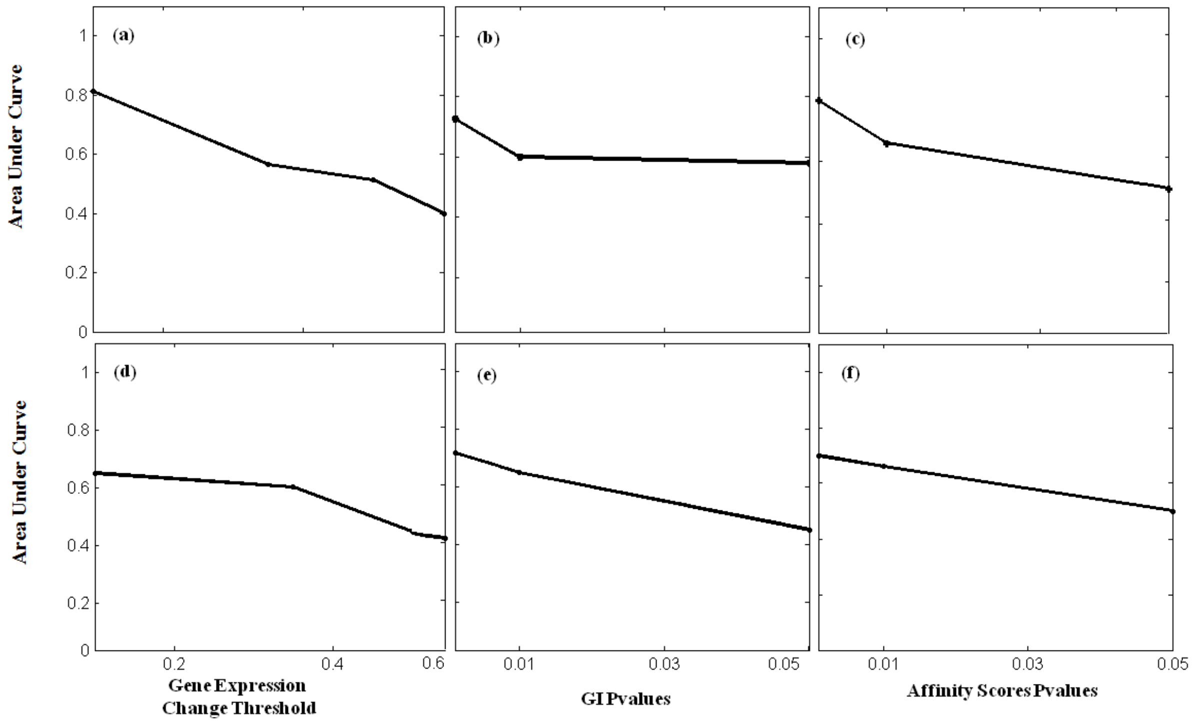

Figure S2: Performance of mTRIM on the Harbison dataset using (a) different gene expression cutoffs, (b) different GI cutoffs, (c) different Afnscore cutoffs; and performance of mTRIM on the Reimand dataset using (d) different gene expression cutoffs, (e) different GI cutoffs, (f) different Afnscore cutoff.

Table S1: Yeast cell cycle time series data. Three time-series microarray datasets commonly used for yeast cell cycle studies were collected (Spellman 1998). It has in total 49 time-points. In these studies, yeast cells were synchronized to the same cell cycle stage, released from synchronization, and then total RNA samples were taken at even intervals for a period of time.

Data type	Time-points
A-factor synchronization	Every 7 minutes for 2 hours
CDC-28 heat-based synchronization.	Every 10 minutes for 2.67 hours.
Elutriation synchronization.	Every 30 minutes for 6.5 hours.

Table S2: Number of the regulatory modules on both Harbison and Reimand datasets. Clearly, in the majority of the regulatory modules, target genes are regulated by multiple TFs.

Number of TFs in a regulatory module	Number of regulatory modules on the Harbison Dataset	Number of regulatory modules on Reimand Dataset
1	988	630
2	504	253
3	296	82
4	153	36
5	94	16
6	34	6
7	23	6
8	29	1
9	8	0
10	15	1
11	8	0
12	8	0
13	3	0
14	3	0
15	0	0
16	2	0
17	1	0
18	1	0
19	2	0

References

Spellman, P. et al. (1998). Comprehensive Identification of Cell Cycle-regulated Genes of the Yeast Saccharomyces cerevisiae by Microarray Hybridization. Molecular Biology of the Cell, 9, 3273-3297.
